# Critical Evaluation of Treatment Response, Driver Mutations, and Circulating Tumor DNA as Markers of Tumor Biology in Colorectal Liver Metastasis

**DOI:** 10.3390/cancers18071111

**Published:** 2026-03-30

**Authors:** Mikel Madi, Antony Haddad, Kyoji Ito, Neal Bhutiani, Jean-Nicolas Vauthey

**Affiliations:** 1 Department of Surgical Oncology, The University of Texas MD Anderson Cancer Center, Houston, TX 77030, USA; mmadi@mdanderson.org (M.M.);; 2 Department of Surgery, University of Louisville, Louisville, KY 40292, USA; 3 Department of Microbiology and Immunology, University of Louisville, Louisville, KY 40292, USA

**Keywords:** colorectal liver metastasis, tumor biology, genetic mutations, chemotherapy response, circulating tumor DNA

## Abstract

Background: Improved assessment of tumor biology has contributed to better outcomes in colorectal liver metastasis (CLM). Previously, tumor biology was assessed based on clinical factors such as number and size of metastases, primary tumor characteristics, and extent of extrahepatic disease. Currently, tumor biology assessment includes response to chemotherapy, genetic mutations, and circulating tumor DNA (ctDNA). Methods: A review of the literature in Medline/Pubmed, Embase, and Cochrane Library was conducted using keywords and MeSH terms. Results: Tumor response to chemotherapy can be assessed using pathologic and radiologic criteria. Radiologic morphologic response has been associated with more accurate determination of outcomes compared with size-based criteria. Pathologic tumor response can be assessed by the percentage of cancer cells remaining within each tumor, the ratio of cancer cells to fibrosis, and the thickness of the tumor–normal liver interface. Six driver mutations are consistently associated with outcomes in CLM: *RAS/BRAF*, *TP53*, *SMAD4*, *FBXW7*, and *APC*. All are associated with decreased overall survival (OS) and recurrence-free survival (RFS) except for *APC*, which is associated with better survival. More than 50% of patients have co-mutations, and a three-tier pathway-centric risk score integrating these mutations offers a more comprehensive approach. While mutations should be considered when evaluating for locoregional therapy, it should not influence ablation margins, surgical margins, or parenchymal sparing approach. Preoperative ctDNA is associated with worse survival, but clearance after hepatectomy is associated with improved survival. Postoperative ctDNA status is associated with recurrence and has the potential to guide the choice of adjuvant chemotherapy. Conclusion: Tumor biology enables informed, precise, and personalized decision-making. Integration of response to chemotherapy, driver mutations, and ctDNA into routine practice is critical to improve CLM management.

## Simple Summary

Assessment of tumor biology has become increasingly relevant in the management of colorectal liver metastasis (CLM). Tumor biology reflects tumor aggressiveness and was previously assessed using clinical features including tumor size, number, and extent. More recently, tumor biology has expanded to include response to chemotherapy, genetic mutations, and tumor-specific biomarkers. This review explains the different approaches available for assessing tumor response to chemotherapy. In addition, we summarize the current evidence on the role of driver mutations in prognosis and informing liver-directed therapies. We discuss the role of circulating tumor DNA (ctDNA) as a true biomarker in CLM treatment and its future applications. Finally, we highlight the use of tumor biology for risk stratification, operative decision-making, and perioperative treatment strategies. Integrating tumor biology into routine clinical practice represents a critical step toward personalized management of CLM.

## 1. Introduction

More than half of patients with colorectal cancer (CRC) present with liver metastasis [1]. Over the past two decades, long-term outcomes for patients with colorectal liver metastasis (CLM) have markedly improved, with 5-year overall survival (OS) exceeding 50% [2]. This is due to increased adoption of curative-intent approaches, advancements in surgical techniques, development of targeted systemic therapies, and personalized treatment decision-making based on tumor biology [3].

Tumor biology was classically assessed using number and size of metastases, primary tumor characteristics, extent of extrahepatic disease, and tumor markers [4]. More recently, assessment of tumor biology has evolved to include tumor response to chemotherapy, genetic mutations, and circulating tumor DNA (ctDNA) [5]. Currently, CLM tumor biology is a cornerstone for prognostic assessment, defining resectability, and determining indications for transplantation [6].

In this review, we discuss three aspects of CLM tumor biology: radiologic and pathologic approaches to assessing tumor response to chemotherapy, the impact of genetic mutations on long-term outcomes and local therapy, and the role of ctDNA in guiding personalized management.

## 2. Methods

A narrative review of the literature was performed using the Medline/PubMed, Embase, and Cochrane Library databases, up to October 2025, using MeSH terms and keywords. The terms “Colorectal Liver Metastasis”, “Colorectal Liver Metastases”, “Colorectal Liver Mets”, “Colon Cancer Metastasis to the Liver”, “CLM”, and “CRLM” were searched in the title and/or abstract. Only English language articles were considered for screening. No restrictions were made on publication date. All pertinent articles’ references were reviewed to identify additional relevant studies.

## 3. Response to Chemotherapy

Less than 20% of CLM are candidates for upfront resection, and around 40% of initially unresectable patients undergo resection after systemic chemotherapy [7,8]. Nowadays, most patients receive preoperative chemotherapy mainly consisting of FOLFOX or FOLFIRI, with or without anti-VEGF (vascular endothelial growth factor) and/or anti-EGFR agents [9]. It is crucial to evaluate tumor response to chemotherapy to guide further management, avoid unnecessary side effects, and reduce extra costs [10].

### 3.1. Radiologic Response

Given its availability, noninvasive nature, and relatively low cost, radiologic assessment based on computed tomography (CT) has played an essential role in CLM treatment. It is intuitive to evaluate tumor response to chemotherapy based on metastasis size, with tumor shrinkage indicating a good response. This assessment was first quantified by the World Health Organization (WHO) criteria, which relies on the tumor volume based on longest perpendicular and horizontal diameters multiplication (Table 1) [11]. Recently, a simpler single-parameter Response Evaluation Criteria in Solid Tumor (RECIST) approach was introduced based only on the diameter of the largest tumor, classifying patients into four groups (complete response, partial response, progressive disease, and stable disease) (Table 1) [12]. Despite its simplicity, this approach has many limitations. First, relying on tumor diameter is not representative of the volume, since most tumors are not spherical [13]. Furthermore, the shrinkage may occur in a non-uniform fashion without affecting the longest diameter [14]. In contrast to cytotoxic chemotherapy, many antineoplastic agents are cytostatic, such as bevacizumab and cetuximab, leading to cell growth arrest without directly killing tumor cells. Despite their proven long-term advantages, these drugs were not found to be associated with objective radiologic response based on the RECIST criteria [15].

**Figure 1:**
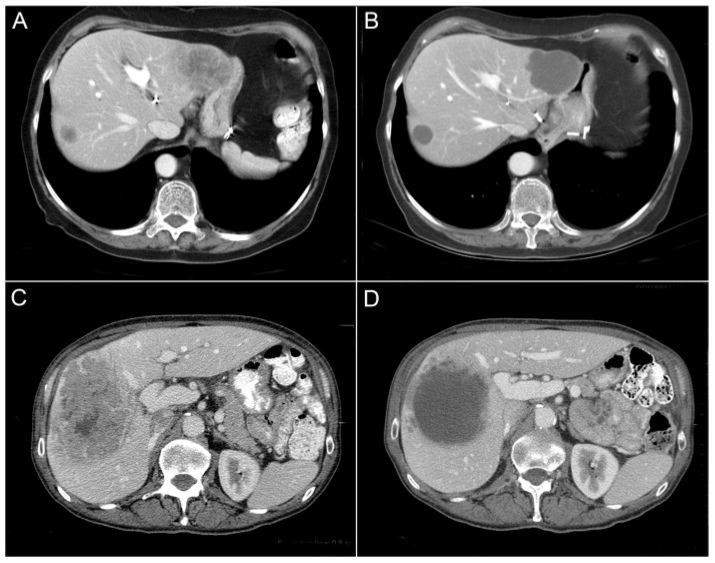
Pre- and post-treatment CT scans showing RECIST stable disease and morphologic optimal response (**A**,**B**), characterized by homogeneous attenuation and sharp tumor–liver interface; morphologic incomplete response (**C**,**D**), with homogeneous attenuation but ill-defined tumor–liver interface remaining after treatment. Adapted from Chun et al. [10] with permission.

These limitations of the RECIST criteria prompted better radiologic assessment. Many radiologists observed changes in tumor morphology without significant size reduction after targeted chemotherapy, especially with bevacizumab [10]. Chun et al. described these morphological changes as homogenous and low attenuation with a thin, sharply defined tumor–liver interface (Figure 1) [10]. Based on these changes, the radiologic morphologic criteria was put forth, stratifying patients into three groups irrespective of tumor size change (optimal response, incomplete response, and no response) (Table 1) [10]. Importantly, Shindoh et al. validated this criteria in 209 patients and showed that the morphologic criteria is sufficiently associated with OS regardless of the chemotherapy regimen [19]. This study showed no correlation between morphologic and RECIST criteria [19]. Nishioka et al. validated this association with OS and RFS on a Japanese cohort [20]. Similarly, Mazard et al. reported comparable outcomes in patients treated with bevacizumab-based chemotherapy for unresectable CLM. Notably, the RECIST criteria was not associated with RFS in this study [21]. Overall, RECIST provides a standardized and objective size-based assessment, whereas morphologic criteria better capture tumor appearance changes (Table 2). Therefore, integration of morphologic criteria and size criteria may be needed to improve accuracy of radiologic response to chemotherapy in patients with CLM [19].

### 3.2. Pathologic Response

On pathologic evaluation, untreated tumors are characterized by a mixture of viable tumor cells and necrosis. After exposure to chemotherapy, the cellular response is manifested by tumor cell death associated with fibrosis and mucin deposition, constituting the basis of chemotherapy response assessment [26]. This classifies patients into three groups (complete response, major response, and minor response) based on the mean of the percentage of cancer cells remaining within each tumor (Table 1) [16]. In a cohort of 305 patients who underwent irinotecan- or oxaliplatin-based preoperative chemotherapy regimens, Blazer et al. reported a significant difference in 5-year OS based on pathologic response (75% in complete response, 56% in major response, and 33% in minor) [16].

Another reliable approach to assess CLM response to chemotherapy is the tumor regression grade (TRG) scoring system, which relies on the ratio of cancer cells to fibrosis, classifying patients into five groups (Table 1). Rubbia-Brandt et al. showed that partial histological response (TRG3) and no histological response (TRG4 and 5) were associated with significant decrease in 3-year and 5-year OS compared with major histological response (TRG1 and 2) [17]. The majority of residual cancer cells post-chemotherapy aligns near the tumor–normal liver interface (TNI); thus, measuring tumor thickness at this interface was also reported as a tool to assess pathologic response in CLM (Table 1) [18]. In a cohort of 171 patients, increased TNI thickness was independently associated with a stepwise decrease in 5-year RFS between TNI < 0.5 mm (58%), TNI 0.5–5 mm (24%), and TNI ≥ 5 mm (11%) [27].

### 3.3. Radiologic–Pathologic Correlation

Despite differences in approach, both radiologic and pathologic measures were found to be reliable in assessing CLM response to chemotherapy [28]. In fact, studies have shown a correlation between both approaches, further validating their relevance. Maru et al. reported an excellent correlation between tumor thickness at TNI and radiologic response by morphologic criteria; however, a weak correlation was seen between tumor thickness at TNI and RECIST criteria [18]. Both RECIST and morphologic criteria were associated with the percentage of residual tumor cells on pathologic assessment [10]. Based on a cohort of 234 patients, Chun et al. reported a median residual tumor percentage of 20%, 50%, and 70% for morphologic non-responders, suboptimal responders, and optimal responders, respectively [10]. The median percentages of residual tumors were 30%, 50%, and 70% for RECIST partial response, stable disease, and progressive disease, respectively [10]. Shindoh et al. confirmed these findings with an emphasis on the superiority of morphologic criteria over RECIST in correlating with the pathologic response. Major pathologic response was present in 92% of patients with optimal morphologic response, in contrast to 83% of patients with partial response by RECIST criteria [19].

Despite the clinical utility of treatment response assessment in CLM, several limitations must be acknowledged. Notably, in the CAIRO5 trial, morphologic response was not associated with OS, and neither RECIST nor morphologic response was associated with recurrence [29]. Furthermore, morphologic and pathologic responses across multiple metastases in the same patient are often combined into a single category, without fully accounting for intrapatient tumor heterogeneity [29]. A major challenge in imaging-based assessment is disappearing liver metastases after chemotherapy, which occurs in approximately 20% of lesions and does not always correspond to a complete pathologic response [30]. Pathologic response assessment is only available postoperatively and therefore cannot inform preoperative decision-making. Moreover, chemotherapy-associated liver injury may further complicate pathologic evaluation, as sinusoidal obstruction syndrome has been associated with reduced pathologic response [31].

## 4. Genetic Mutations

Initial studies on genetic mutations in CLM aimed to understand the biological relationship between the primary tumor and metastatic disease [32]. These mutations were then used by medical oncologists to inform systemic therapy selection [33]. Currently, the evaluation of these mutations is critical to determining CLM tumor biology and informing biology-driven clinical management. Six driver mutations in five signaling pathways have been consistently associated with CLM tumor biology: *RAS/BRAF*, *SMAD4*, *TP53*, *APC*, and *FBXW7*, corresponding to the RTK–RAS, TGF-β, p53, Wnt, and Notch pathways, respectively [34].

### 4.1. Frequency, Presentation, and Prognosis

**Figure 2:**
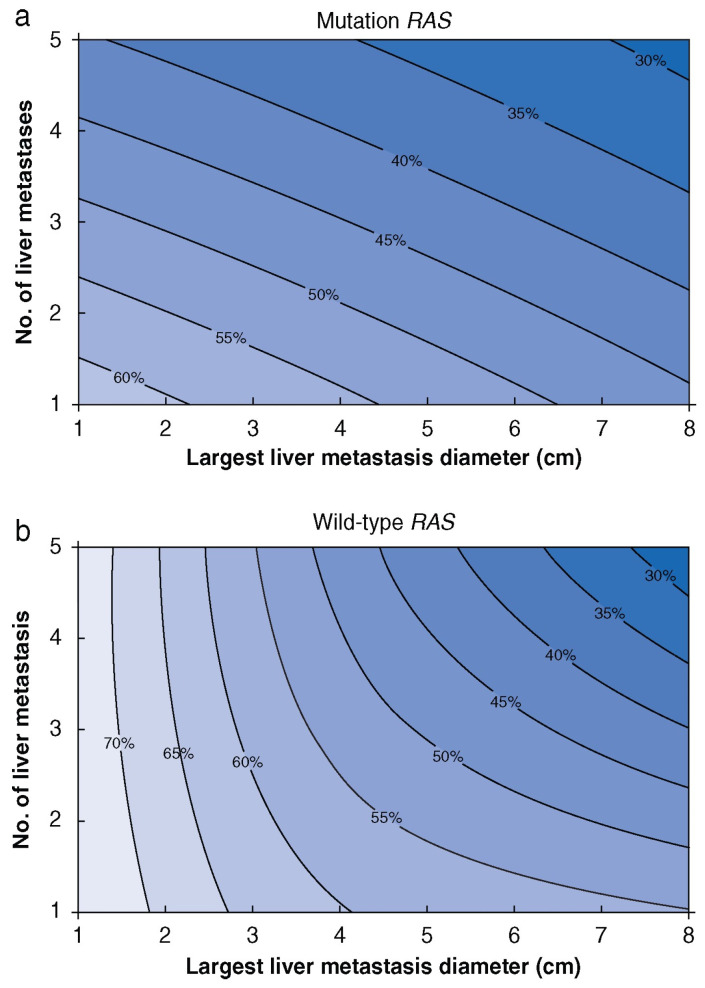
Contour plot of 5-year overall survival probability according to largest diameter and number of colorectal liver metastases for patients with *RAS* mutation (**a**) and *RAS* wild-type (**b**). The color gradient reflects the 5-year overall survival probability, with darker shades indicating lower survival and lighter shades indicating higher survival. Adapted from Kawaguchi et al. [42] with permission.

The *RAS* genes, including *KRAS* and *NRAS*, are proto-oncogenes, and they are part of the mitogen-activated protein kinase (MAPK) signaling pathway, which regulates cellular growth, apoptosis, and angiogenesis [35]. As a downstream effect, *RAS* mutations upregulate metastasis-promoting factors like ANGPT2 and CXCR4 that disturb the cytokine milieu of the tumors, possibly affecting tumor–stroma interactions, favoring liver metastasis formation [36]. *RAS* mutations also promote immune evasion by modulating tumor microenvironment, promoting metastatic dissemination [37]. The association of *RAS* mutation with decreased OS and recurrence-free survival (RFS) in patients with CLM was first reported in 2013 by Vauthey et al. in a cohort of 193 patients [38]. It was detected in 40–50% of patients with CLM and was associated with right-sided primary tumors and synchronous metastases [39,40]. A recent meta-analysis of 36 studies with a total of 15,766 patients supported these findings (Table 3) [41]. Given its clinical importance, Kawaguchi et al. developed an externally validated contour prognostic model based on the number of CLM, the largest tumor diameter, and *RAS* mutation status (Figure 2) [42]. In patients without recurrence two years after CLM resection, *RAS* mutation was the only factor associated with increased risk of recurrence [43]. Thus, these findings have prompted consideration of more intensive surveillance between 2 and 4 years in patients with *RAS* mutation at some high-volume centers such as The University of Texas MD Anderson Cancer Center, although broader validation is still needed before this approach can be generalized [43].

*BRAF* mutation, one of the most deleterious mutations in CRC, is rarely found in resectable CLM due to its aggressive biology and advanced disease at diagnosis [45]. *BRAF* is also a proto-oncogene and functions as another activator of MAPK signaling pathway and a member of the rapidly accelerated fibrosarcoma (RAF) protein kinases family [45]. *BRAF* mutations stimulate glutathione synthesis via upregulation of glutamate–cysteine ligase, which protects tumors from oxidative stress during distant metastasis to the liver and the lungs [46]. Furthermore, *BRAF* mutation may enhance metastasis by regulating CXCL16 expression and promoting angiogenesis in the tumor microenvironment [47]. A multi-institution matched case control study by Gagniere et al., including 1497 patients with CLM, showed that *BRAF* mutation is associated with lower median OS and RFS (Table 3) [48]. V600E *BRAF* mutation is specifically associated with worse survival compared with non-V600E *BRAF* and was reported to be the strongest predictor of OS and RFS in CLM [49]. *BRAF* mutation is also used as a contraindication to transplant for patients with CLM [6].

*TP53* encodes p53, a tumor suppressor protein, and is the most commonly mutated gene in CLM, with a prevalence of up to 77% [34]. It plays a major role in maintaining genomic stability by regulating cell cycle arrest and apoptosis following DNA damage, and its mutations allows for proliferation of unstable tumor cells [50]. *TP53* mutation is shown to negatively impact both long-term oncological outcomes, and its effect is more pronounced in the presence of *RAS/TP53* co-mutation (Table 3) [34]. Furthermore, *TP53* mutation is independently associated with worse pathologic response to chemotherapy, reflecting more aggressive tumor biology [51].

*SMAD4* is a tumor suppressor gene in the TGF-β signaling pathway, which normally regulates cell differentiation and growth inhibition [52]. When *SMAD4* is lost, TGF-β signaling shifts from tumor-suppressive to pro-metastatic through *SMAD*-independent pathways, particularly Rho/ROCK/LIMK pathways [53]. Furthermore, loss of *SMAD4* promotes the accumulation of myeloid-derived suppressor cells through the CCL15–CCR1 chemokine axis, which facilitates invasion and creates an immunosuppressive microenvironment [54,55]. *SMAD4* mutation is present in 13% of patients with CLM and is associated with worse oncological outcomes [56]. In a cohort of 278 patients with CLM, *SMAD4* mutation was associated with significantly lower 3-year OS after hepatectomy [56]. These findings were validated in an external cohort of patients with stage IV rectal cancer treated with chemotherapy alone [56]. A meta-analysis including a total of 3020 patients also reported an increase in HR for OS and RFS in patients with *SMAD4* mutation (Table 3) [41].

Recently, an additional mutation has been associated with poor prognosis in patients with CLM. *FBXW7* mutations disrupt ubiquitin-mediated degradation of oncoproteins and cell cycle regulators within the Notch pathway, contributing to uncontrolled proliferation and metastatic potential [57]. Around 6% of patients with CLM have *FBXW7* mutation, independently associated with decreased OS (Table 3) [44]. A similar association was observed in CRC patients based on a meta-analysis of ten studies including 4199 patients [58].

*APC* functions as a tumor suppressor gene by degrading β-catenin and regulating the WNT pathway [59]. Unlike the previously mentioned mutations, *APC* mutation is associated with improved oncologic outcomes in mCRC [60]. A study of 579 patients with CLM showed that *APC* mutation is independently associated with better OS (Table 3). These findings were validated in an external cohort of patients with unresectable metastatic CRC, with a comparable OS HR [34].

### 4.2. Co-Mutations and Pathway-Centric Risk Classification

**Figure 3:**
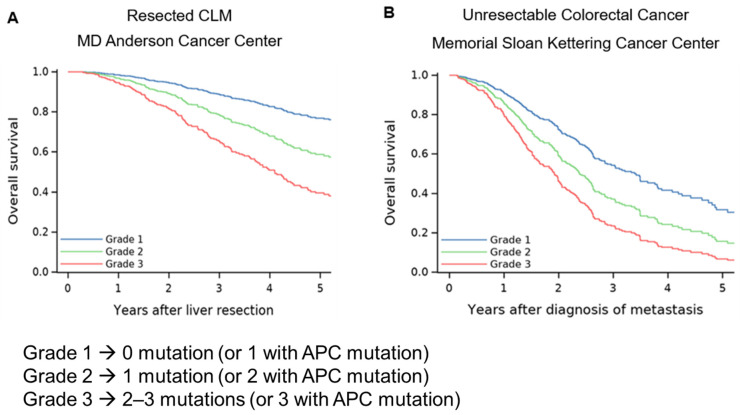
Overall survival (OS) based on the number of mutations. (**A**) OS in 561 patients who underwent resection of CLM at MD Anderson Cancer Center. OS curves after adjustment for age, primary lymph node metastasis, number of CLM, and largest CLM diameter. (**B**) OS in 503 patients with unresectable CLM at Memorial Sloan Kettering Cancer Center. OS curves after adjustment for age and number of sites involved at initial diagnosis of metastasis. Adverse mutations listed as: *RAS/BRAF*, *TP53*, and *SMAD4*. Adapted and modified from Kawaguchi et al. [34] with permission.

More than 50% of patients with CLM harbor mutations in multiple genes, with some tumors carrying up to four driver mutations [34]. Due to its synergistic deleterious effect on oncologic outcomes, the *RAS/TP53* co-mutation is the most studied for patients with CLM [61,62]. In a cohort of 401 patients with CLM, Chun et al. showed that *RAS/TP53* co-mutation is present in 31.4% of patients and is independently associated with decreased OS [63]. Moreover, Maki et al. reported that *KRAS* mutation is associated with worse outcomes only when *TP53* is co-mutated [64]. Interestingly, in a cohort of 507 patients, there was no difference in OS and RFS between patients with *RAS* mutation but wild-type *TP53* and *SMAD4* compared with patients with *RAS* wild-type [65]. Thus, a comprehensive multigene assessment of mutations is more reflective of tumor biology. Kawaguchi et al. developed a robust risk stratification model for resectable and unresectable metastatic colorectal cancer, accounting for the four most commonly altered pathways: *TP53*, *APC*, *RAS*/*BRAF*, and *SMAD4*. This pathway-centric risk classification stratifies patients into three grades based on their genetic profile. Grade 1 includes patients with no driver mutations or one adverse mutation in the presence of *APC*. Grade 2 includes patients with one adverse mutation or two adverse mutations in the presence of *APC*. Grade 3 includes patients with two or more adverse mutations or three driver mutations in the presence of *APC*. The covariate-adjusted 5-year OS is significantly higher in grade 1 (76.9%), compared with grade 2 (58.7%), which was higher than grade 3 (39.5%) (Figure 3) [34]. Another three-gene scoring system based on *RAS*, *SMAD4*, and *APC* mutational status was proposed in a Chinese cohort and showed consistent association with OS in patients with CLM [66].

### 4.3. Local Therapy Considerations

In addition to its prognostic value, tumor biology is also considered in guiding the selection and extent of local therapy for CLM. Odisio et al. showed that *KRAS* mutation is associated with an earlier and higher rate of local tumor progression after CLM ablation, implying that a 5 mm margin can be considered safe for *KRAS* wild-type, but a margin of 10 mm is needed when *KRAS* is mutated [67]. A subsequent follow-up study by the same group, using deformable CT image registration and autosegmentation, showed that a margin ≥ 5 mm was not associated with local tumor progression, irrespective of *RAS* mutational status [68].

Given its association with a worse overall prognosis, some investigators have linked *KRAS* mutation with positive surgical margins and increased local recurrence risk [69,70]. However, a recent meta-analysis of 19 studies and a total of 7391 patients did not support this association [71]. Some researchers argued that more extensive surgery, such as anatomic resection, is needed in patients with *KRAS* mutation, given their aggressive biology [72]. However, a propensity score-matched study of 622 patients with CLM showed that OS, RFS, and liver-specific RFS were similar between anatomic and non-anatomic resections for both mutated and wild-type *KRAS* patients [73]. Furthermore, in a cohort of 415 patients, Nishioka et al. showed that local recurrence is not associated with *RAS/TP53*, *BRAF*, *SMAD4*, or *FBXW7* mutations [74]. More importantly, this study showed that neither driver mutations nor R1 resection are associated with an increase in local recurrence risk after R0 curative intent hepatectomy [74].

Taken together, these findings suggest that tumor biology alone should not determine surgical margins, and an R0 resection should always be attempted [75,76]. Given the increased risk of recurrence in patients undergoing curative intent treatment of CLM, the extent of resection should take into account the feasibility of repeat curative intent treatment in case of recurrence. For instance, parenchymal sparing hepatectomy (PSH) allows for increased salvageability in case of liver recurrence for patients with solitary small tumors [77]. Furthermore, PSH is not associated with increased recurrence despite the associated higher rate of R1 resection in patients with bilateral extensive CLM [78]. Accordingly, PSH is the recommended surgical approach for patients with CLM [75].

*BRAF* mutation is associated with unresectable CLM at presentation and lower rates of conversion to resectability after systemic therapy [79,80]. However, *BRAF* mutation alone is not considered a contraindication for surgery in resectable CLM [81]. When evaluating liver transplantation (LT) in unresectable cases, *BRAF* has been proposed as a contraindication to LT given its aggressive tumor biology [82]. A review of the ongoing clinical trials on LT in CLM reported that eight ongoing trials have excluded patients with *BRAF* mutation [83].

The clinical significance of somatic mutations in CLM is increasingly recognized, but several limitations remain present. Most of the discussed evidence is based on high-volume tertiary referral centers, potentially limiting the generalizability of these findings at the current timepoint. However, meta-analyses evaluating the effect of individual mutations on long-term survival have shown low-to-moderate heterogeneity across studies [41]. Although pathway-centric risk classification represents a more comprehensive and externally validated approach, it would still benefit from further validation across diverse cohorts. Finally, the clinical applicability of mutational profiling remains constrained by substantial cost and resources, limiting its availability across centers.

## 5. Circulating Tumor DNA

Recurrence rates in CLM remain high, exceeding 50%, and mostly occurring within the first two years [43]. Strategies for prediction or early detection of recurrence are highly emphasized for optimal treatment of patients with CLM. Well-established tumor markers such as CEA are helpful, especially when their dynamic response to preoperative chemotherapy and after CLM resection is studied [84]. Recently, ctDNA has emerged as an accurate biomarker in CRC, with promising applications in prognosis, response to systemic therapy, and early recurrence detection [85,86].

**Figure 4:**
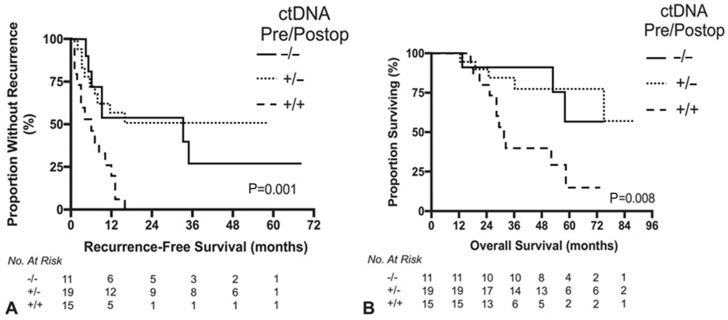
Kaplan–Meier survival analyses by perioperative ctDNA dynamics among patients who underwent curative intent surgical clearance of metastatic CRC including hepatectomy. (**A**) Recurrence-free survival. (**B**) Overall survival. Log-rank *p* values. Prehep/Postop indicates prehepatectomy/postoperative. Adapted and modified from Newhook et al. [93] with permission.

Serial ctDNA measurements may serve as a sensitive dynamic indicator of disease burden and treatment response [87]. For instance, a significant decline in ctDNA as early as four weeks after treatment initiation may help identify patients who are responding well to systemic therapy, whereas persistently elevated or rising ctDNA levels during treatment may suggest treatment resistance and may prompt consideration of a change in regimen [88]. In addition, longitudinal ctDNA sequencing may detect emergent actionable resistance mutations during active treatment, which could further inform therapy selection [89]. Furthermore, several studies have associated ctDNA status prior to liver resection with long-term survival in patients with CLM. Based on a cohort of 212 patients with solitary CLM, Kobayashi et al. reported that detection of ctDNA prior to hepatectomy was associated with significantly decreased RFS [90]. Furthermore, the detection of specific genes such as *KRAS*, and *TP53* in preoperative ctDNA was associated with worse survival [91,92]. However, Newhook et al. showed that clearance of ctDNA after surgery is associated with outcomes comparable with those with undetectable preoperative ctDNA and that sustained postoperative ctDNA detection is associated with worse OS and RFS (Figure 4) [93].

**Figure 5:**
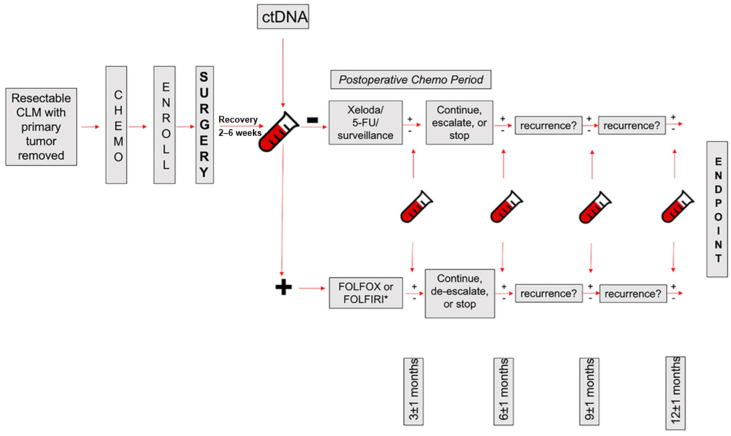
NCT05062317: Risk-stratified post-hepatectomy chemotherapy for patients with resectable colorectal liver metastases. Arrows indicate the direction of clinical management and follow-up over time, while symbols denote ctDNA sampling time points. Adapted from Adams et al. [99] with permission.

After curative intent surgery, detection of ctDNA without any radiographic evidence of disease is termed minimal residual disease (MRD) and is associated with increased risk of recurrence [94]. In a cohort of 105 patients, Nishioka et al. showed that detection of ctDNA within 180 days after hepatectomy is associated with worse median RFS (6.3 months vs. 12.2 months) [95]. This study also showed that *RAS/TP53* co-mutation is associated with increased risk of ctDNA detection postoperatively [95]. Furthermore, Marmorino et al. corroborated these findings by showing that postoperative detection of ctDNA is associated with significantly worse RFS on multivariate analysis [96]. At present, a detectable ctDNA postoperatively is not sufficient to start adjuvant chemotherapy (ACT) in patients with resected CLM. However, the GALAXY trial reported that ctDNA detection following the resection of stage II-IV CRC can identify patients who benefit from ACT based on an analysis of 644 patients [97]. In the same cohort, longitudinal assessment of ctDNA during ACT showed that sustained clearance is associated with better 2-year RFS (89.0% vs. 3.3%) and OS (100% vs. 82%) compared with transient clearance [98]. These findings constitute the basis of an ongoing clinical trial at the University of Texas MD Anderson Cancer Center (NCT05062317) using ctDNA to guide the ACT selection following CLM resection. This trial classifies patients into two groups based on ctDNA status several weeks after hepatectomy. This trial aims to evaluate whether ctDNA negative patients can safely receive a less intensive systemic therapy regimen than their ctDNA positive counterparts without increasing risk of recurrence (Figure 5). ctDNA shows considerable potential with several clinical applications, and further studies are needed to fully elucidate its role in CLM management.

While ctDNA has demonstrated strong prognostic value for detecting MRD and predicting recurrence after CLM resection, several shortcomings limit its clinical use. Most available studies are still limited by relatively small sample sizes [93,94]. A major challenge in ctDNA-based detection of MRD is the analytical sensitivity of current assays, with false-negative results being reported in up to 33%, particularly with tumor-agnostic platforms [94]. Furthermore, ctDNA is mostly interpreted as a binary variable (detectable vs. undetectable); however, further study of ctDNA as a continuous variable may further refine prognostication [93]. In addition, the costs associated with repeated testing further limits studies evaluating longitudinal ctDNA dynamics and its full clinical potential.

## 6. Future Directions in CLM Biology

Beyond the main biologic pillars discussed in this review, several emerging domains are likely to further refine CLM management. A deeper understanding of the tumor microenvironment, including its role in tumor initiation, progression, and metastasis, may uncover new therapeutic endeavors [100]. Clonal evolution is also gaining increasing interest in mCRC, as detection of emerging resistant clones may be helpful for adapting therapies [101]. Likewise, further insight into intrapatient tumor heterogeneity is needed to better understand its influence on tumor development, treatment response, and disease outcomes [102]. Multi-omics approaches integrating genomic, transcriptomic, proteomic, and epigenomic data also hold promise for more advanced treatment strategies [103]. In addition, investigation of hepatocyte metabolic reprogramming may reveal liver-specific mechanisms of metastasis [104]. Lastly, artificial intelligence may help integrate complex datasets to uncover patterns not detectable through conventional analyses, ultimately supporting more personalized patient care [105].

## 7. Conclusions

Tumor biology for CLM has been evolving to include more nuanced variables. Radiologic and pathologic assessments beyond size reduction better portray the effects of targeted therapies and tumor response. Somatic gene mutations reflect the tumor pathophysiology and help predict aggressiveness. ctDNA reflects ongoing tumor status and aids early detection of MRD and recurrence. Together, these domains support a more biology-informed approach for patient selection, perioperative decision-making, surveillance, and prognostication in CLM. Further validation of the current evidence across diverse patient populations is needed to broaden its applicability. Studies with in-depth genetic analysis and ctDNA dynamics may contribute to further advancing the field by improving oncologic risk stratification and patient outcomes.

## Figures and Tables

**Table 1:** Radiologic and pathologic criteria used to assess colorectal liver metastasis response to chemotherapy.

	Criteria	Assessment Parameter	Classification
Radiologic	WHO [11]	Decrease in tumor volume	Complete response: disappearance of all known disease Partial response: ≥50% decrease in total tumor load Progressive disease: ≥25% increase in the size of one lesion, emergence of new lesions No change: neither partial response nor progressive disease
	RECIST [12]	Decrease in tumor largest diameter	Complete response: disappearance of all known disease Partial response: ≥30% decrease in the sum of diameters of all lesions Progressive disease: ≥20% increase, emergence of new lesions, or unequivocal increase in diameter of non-target lesions Stable disease: neither partial response nor progressive disease
	Morphologic [10]	Lower attenuation, More homogeneous content Sharper tumor–normal liver	Optimal response: a very pronounced change, like a hepatic cyst in the portal phase Incomplete response: subtle morphological changes, not reaching a pseudocyst appearance No response: no morphological changes between the pre-treatment and post-treatment imaging
Pathologic	Tumor viability [16]	Percentage of residual cancer cells in the total tumor area	Complete response: no residual cancer cells Major response: 1% to 49% residual cancer cells Minor response: ≥50% residual cancer cells
	Tumor regression grade [17]	Ratio of cancer cells to fibrosis	TRG1: no cancer cells and major fibrosis TRG2: rare cancer cells dispersed through pronounced fibrosis TRG3: more cancer cells in the setting of predominant fibrosis TRG4: residual cancer cells predominating fibrosis TRG5: major residual cancer cells with minimal to no fibrosis
	Tumor thickness [18]	Thickness of tumor–normal liver interface (TNI)	TNI < 0.5 mm TNI 0.5–5 mm TNI ≥ 5 mm

Abbreviations: WHO: World Health Organization; RECIST: response evaluation criteria in solid tumor; TRG: tumor regression grade; TNI: tumor–normal liver interface.

**Table 2:** Comparison of advantages and limitations of RECIST and morphologic criteria for response assessment in colorectal liver metastases.

	RECIST	Morphologic
Assessment	Unidimensional tumor size measurements	Qualitative changes in tumor appearance including attenuation, enhancement, and tumor–liver interface
Advantages	- Objective quantitative measurements - Ease of implementation - Standardized and widely accepted indicator of response [19] - High interobserver agreement [22]	- Superior correlation with pathologic response [23] - Superior correlation with OS regardless of chemotherapy regimen [19] - Reflection of tumor biology (cell viability) [10] - High interobserver agreement [10]
Limitations	- Poor assessment of response to bevacizumab [10] - Inability to capture qualitative tumor changes - Inconsistent association with survival and pathologic response [21] - Strict cutoff values limiting discriminatory ability [24]	- Subjective assessment requiring experienced radiologists - Dependent on high-quality CT imaging and adequate enhancement - Challenging for small tumors (<1–1.5 cm) [19] - Limited validation with MRI [25]

**Table 3:** Frequency of the six driver mutations in colorectal liver metastases and their effect on overall survival and recurrence-free survival.

Mutation	Pathway	Frequency	OS HR	RFS HR
*RAS*	RTK–RAS	34.2%	1.68 (1.54–1.84) ^a^	1.46 (1.33–1.61) ^b^
*BRAF*	RTK–RAS	4.8%	2.62 (2.14–3.20) ^c^	1.89 (1.32–2.73) ^d^
*TP53*	p53	77.2%	1.88 (1.3–2.74) ^e^	-
*SMAD4*	TGF-β	11.5%	1.93 (1.56–2.38) ^f^	1.95 (1.31–2.91) ^g^
*FBXW7*	Notch	5.7%	1.99 (1.15–3.45) ^h^	-
*APC*	Wnt	76.7%	0.66 (0.49–0.89) ^i^	-

Abbreviations: OS: Overall survival; HR: hazard ratio; RFS: recurrence-free survival. ^a^ Data from a meta-analysis including 36 studies and a total of 15,766 patients [41]. ^b^ Data from a meta-analysis including 20 studies and a total of 8355 patients [41]. ^c^ Data from a meta-analysis including 13 studies and a total of 5831 patients [41]. ^d^ Data from a meta-analysis including 8 studies and a total of 3138 patients [41]. ^e^ Data from a retrospective study including a total of 395 patients [34]. ^f^ Data from a meta-analysis including 5 studies and a total of 222 patients [41]. ^g^ Data from a meta-analysis including 2 studies and a total of 125 patients [41]. ^h^ Data from a retrospective study including a total of 476 patients [44]. ^i^ Data from a retrospective study including a total of 326 patients [34].

## Data Availability

This study is a narrative review of previously published literature. No new data were created or analyzed in this study. Data sharing is not applicable to this article.
